# A Case of Montelukast-Induced Churg-Strauss Syndrome Associated with Liver Dysfunction

**DOI:** 10.1155/2011/412524

**Published:** 2011-07-09

**Authors:** Keiji Matsui, Kosuke Nishijima

**Affiliations:** ^1^Center for Digestive and Liver Disease, Ofuna Chuo Hospital, 6-2-24 Ofuna, Kamakura-city, Kanagawa 247-0056, Japan; ^2^Kamakura Internal Medicine Clinic, 736 Yamanouchi, Kamakura-city, Kanagawa 247-0062, Japan

## Abstract

A 64-year-old woman was admitted to hospital due to protracted diarrhea and liver dysfunction. The patient was diagnosed as Churg-Strauss syndrome (CSS) due to asthma, paranasal sinusitis, hypereosinophilia, and polyneuropathy. There was a history of taking montelukast, a leukotriene receptor antagonist (LTRA), which is thought to have some relationship with CSS. The liver biopsy specimen showed eosinophilic infiltration and centrolobular fatty change. In this paper, we review the relationship between LTRA and CSS. Several lines of evidence suggest that leukotriene plays an important role in maintaining neural tissues. We also review the potential relationship between centrolobular fatty change and pivoxil-containing antibiotics, which was prescribed for sinusitis before admission. Carnitine deficiency induced by pivoxil-containing agents may cause impaired fatty acid oxidation in mitochondria.

## 1. Introduction

Churg-Strauss syndrome (CSS) was first described in 1951 by Churg and Strauss. CSS is a systemic eosinophilic vasculitis with unknown etiology [1]. Typical clinical manifestations include asthma, hypereosinophilia, and neurological symptoms. The most common presenting symptom of neuropathy is a painful paresthesia in the distal legs [2]. Reduced grip strength is also well recognized as early clinical characteristics. Over the past year, there have been several reports of leukotriene-receptor-antagonists-(LTRAs) induced CSS [3, 4]. LTRA is a drug for bronchial asthma. The mechanism by which LTRA treats asthma is to antagonize leukotriene and prevent leukotriene-induced bronchial contraction and fibrosis. The causative role of LTRA on CSS has not yet been proven. Some authors have argued that steroid sparing by LTRA use is responsible for unmasking of a latent CSS [1]. However, there are some reports of LTRA-induced CSS without history of systemic corticosteroids [5–7]. In this paper, we present a case of montelukast-induced CSS associated with liver dysfunction without a previous use of systemic corticosteroids. Montelukast, a kind of LTRA, is widely used for bronchial asthma. The liver biopsy showed eosinophilic infiltration in the portal tract and the liver parenchyma. Centrolobular fatty changes were also seen. This is the first case report of CSS presented with a liver dysfunction and confirmed by the liver biopsy.

## 2. Case Report

A 64-year-old woman suffered from bronchial asthma for ten years and was followed up by a family physician. She had been prescribed montelukast 4 months before. There was no history of oral corticosteroids. Diarrhea and anorexia occurred in the patient a month earlier. The patient noticed her grip strength getting weaker and felt an abnormal sensation in her planters 3 weeks earlier. Cefditoren pivoxil was prescribed for the symptoms of paranasal sinusitis before 2 weeks for 7 days. The patient was referred to our hospital because of the lasting diarrhea and liver dysfunction in laboratory tests. 

Hyperlipidemia and fatty liver had been noted in the previous clinic and atorvastatin calcium had been prescribed for hyperlipidemia. There was no history of alcohol intake. Body mass index was 21.6. On admission, blood tests revealed marked eosinophilia (40% of white blood cells). Laboratory findings also showed an elevation of liver enzymes, immunoglobulin G, immunoglobulin E, erythrocyte sedimentation late, and C-reactive protein. Rheumatoid factor was positive, while antineutrophil cytoplasmic antibodies (ANCAs) and antinuclear antibody (ANA) were negative (Table 1). Although mild liver dysfunction was noted occasionally in the history, transaminase had been less than twice the upper limit of the normal. Transaminase test examined 4 months before admission revealed within normal limits. Acute exacerbation of liver dysfunction was found a few days before admission and it was parallel to increase of eosinophilic counts. On physical examination, there was no wheeze. Indeed, her asthma symptom was well controlled on admission. The patient felt difficulty in walking due to weakness in her legs. Grip strength was 13 kg and 14 kg in the right and left hands, respectively. Painful paresthesia in the distal legs was noted. Detailed neurological examinations including nerve conduction velocity test were not conducted. There was no infiltration in the chest X-ray. 

Magnetic resonance cholangiopancreatography (MRCP) showed no abnormality of the biliary tract. The diagnosis of CSS was made due to asthma, paranasal sinusitis, neurological symptoms, and a marked eosinophilia. 

**Figure 1:**
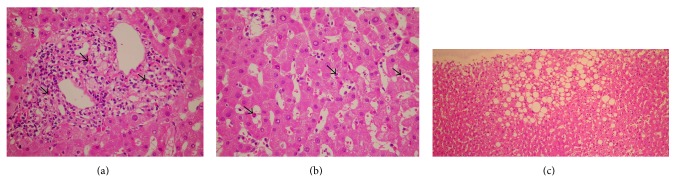
(a, b) In the portal tract, there were mild inflammatory infiltrations with slightly increased eosinophils. In the lobule and the sinusoid, slightly increased eosinophils were also observed (arrow: eosinophils). (c) Besides eosinophilic infiltration, macrovesicular steatosis existed in the centrolobular areas.

The liver biopsy conducted 3 days after admission revealed preserved structures of the lobule. In the portal tract, there were mild inflammatory infiltrations with slightly increased eosinophils. No fibrosis was seen. In the lobule and the sinusoid, slightly increased eosinophils were also observed. A cluster of eosinophils was occasionally seen in the lobule. Besides eosinophilic infiltration, macrovesicular steatosis existed in the centrolobular areas. Neither granuloma nor necrotizing vasculitis was seen (Figures 1(a)–1(c)). 

Montelukast was discontinued immediately after the diagnosis of CSS. We prescribed methylprednisolone 125 mg/day for 3 days. Subsequently, oral prednisolone 40 mg/day was administered. All clinical symptoms and abnormalities in the laboratory tests resolved after these treatments. Prednisolone was thereafter tapered gradually without recurrence of CSS symptoms.

## 3. Discussion

Although asthma is one of the presenting symptoms in CSS, asthma symptom was well controlled in this case at the onset of the neuropathy. Hattori et al. reported that, in most cases, asthma is well controlled when the neurological symptoms develop [2]. Therefore, this finding suggests the difference in the mechanisms of the asthma and the neuropathy. We reviewed the potential contribution of LTRA on neuropathies and found several interesting reports. Leukotrienes are generated from arachidonate by the enzyme 5-lipoxygenase. The biological activities of leukotrienes are bronchial constriction, ileal constriction, fibrogenesis, and several other inflammatory processes. Various inflammatory agents are known to be essential for wound repair and tissue regeneration [8]. Green et al. reported that cyclooxygenase-derived prostaglandins and lipoxygenase-derived leukotrienes coordinately regulate wound closure [9]. Nonsteroidal anti-inflammatory drugs (NSAIDs), inhibitor of cyclooxygenase, cause erosion or ulceration on gastrointestinal membranes. Such a paradoxical inflammatory reaction caused by anti-inflammatory agents is due to overzealous inhibition of prostanoid production required for maintaining the gastrointestinal blood flow. Alternatively, there might be a vulnerable tissue to inhibition of leukotrienes, the other metabolites of arachidonic acids. Although there is no valid evidence to substantiate that peripheral nerves are vulnerable to inhibition of lipoxygenase-leukotriene system, several reports support the relationship between them. First, leukotrienes mediate fibroblast growth factor generation from macrophage [10], and fibroblast growth factor is one of the pivotal agents for axonal regeneration [11]. Second, a lipoxygenase inhibitor AA861 inhibits neurite outgrowth in neural cells *in vitro* via inhibition of F-actin function [12]. Third, Classen et al. showed that a lipoxygenase inhibitor CGS21595 causes axonal degeneration and reduction of grip strength in rats [13], which are remarkably similar to the peripheral nerve damages in CSS [2]. However, the potential role of leukotrienes on neural repair has hitherto received little attention. Further research is needed to investigate the relationship between leukotriene antagonism and neural damages. 

Neither drug lymphocyte stimulation test (DLST) nor patch test was done in this case. Conen et al. stated that a hypersensitivity reaction to LTRA seems unlikely in CSS, because hypersensitivity reactions tend to cause a leukocytoclastic rather than a granulomatous vasculitis [3].

The liver biopsy specimen was in accordance with eosinophilic inflammation. However, it is not clear to what extent these infiltrations of eosinophils contributed to the liver dysfunction, because of the additional fatty depositions. The fatty change found in the liver specimen was thought to be a preexisting abnormality derived from hyperlipidemia. However, there might be a possible role of pivoxil-containing drugs on liver dysfunction in this case.

In our patient, starvation state was evoked by CSS-derived gastrointestinal dysfunctions, that is nausea and diarrhea. Generally, starvation causes increased accumulation of nonesterified fatty acids (NEFA) in hepatocytes [14]. NEFA are carried to mitochondria with carnitine, a fatty acid transporter. There was a history of taking pivoxil-containing antibiotics (cefditoren pivoxil) before admission. Although pivoxil is widely utilized for increasing oral bioavailability of drugs, it causes carnitine deficiency by binding to carnitine and being excreted as pivoxil-carnitine into the urine. Ito et al. reported that even a short-term (7 days) use of pivoxil-containing antibiotics causes carnitine deficiency in healthy volunteers [15]. Increased NEFA and decreased carnitine might synergistically augment fatty depositions in the centrolobular areas. Thus, the liver dysfunctions seen in this patient may not solely be attributable to eosinophilic infiltrations but also the novel mechanism of “pivoxil and starvation induced steatosis”.

In this paper, we propose two novel insights, namely, leukotriene-antagonism-induced neural degeneration and pivoxil-induced steatosis. Although several lines of indirect evidence support these hypotheses, additional investigations are warranted to confirm them.

## Figures and Tables

**Table 1:** Laboratory findings showed marked eosinophilia (eo), elevation of liver enzymes, immunoglobulin G and E, erythrocyte sedimentation late, and C-reactive protein. Rheumatoid factor (RF) was positive, while antineutrophil cytoplasmic antibodies (ANCA) were negative.

Parameters	Value
White blood cells	11050*/μ*L
Eosinophils	40%
Hemoglobin	12.2 g/dL
Platelets	24.3 × 10^4^/*μ*L
IgG	3695 mg/dL
IgA	193 mg/dL
IgM	50 mg/dL
IgD	1.1 mg/dL
IgE	2880 IU/mL
LDH	323 IU/L
AST	78 IU/L
ALT	90 IU/L
ALP	1048 IU/L
y-GTP	281 IU/L
Total bilirubin	1.7 mg/dL
Total protein	9.1 g/dL
Albumin	3.6 g/dL
BUN	13 mg/dL
Creatinin	0.73 mg/dL
Triglyceride	62 mg/dL
Total cholesterol	150 mg/dL
ESR	135 mm/h
CRP	4.94 mg/dL
c-ANCA	−
p-ANCA	−
Rheumatoid factor	+
